# Functions and Therapeutic Roles of Exosomes in Cancer

**DOI:** 10.3389/fonc.2014.00127

**Published:** 2014-05-27

**Authors:** Jacob A. Tickner, Aaron J. Urquhart, Sally-Anne Stephenson, Derek J. Richard, Kenneth J. O’Byrne

**Affiliations:** ^1^Cancer and Ageing Research Program, Translational Research Institute, Queensland University of Technology, Brisbane, QLD, Australia; ^2^Eph Receptor Biology Group, Translational Research Institute, Queensland University of Technology, Brisbane, QLD, Australia

**Keywords:** exosome, cancer, metastatic niche, chemotherapeutic resistance, biomarker

## Abstract

The role of exosomes in cancer development has become the focus of much research, due to the many emerging roles possessed by exosomes. These micro-vesicles that are ubiquitously released in to the extracellular milieu, have been found to regulate immune system function, particularly in tumorigenesis, as well as conditioning future metastatic sites for the attachment and growth of tumor tissue. Through an interaction with a range of host tissue, exosomes are able to generate a pro-tumor environment that is essential for carcinogenesis. Herein, we discuss the contents of exosomes and their contribution to tumorigenesis, as well as their role in chemotherapeutic resistance and the development of novel cancer treatments and the identification of cancer biomarkers.

## Introduction

There is no doubt that identifying novel methods for improving diagnostic and prognostic monitoring of cancer patients, as well as designing novel cancer therapies has proved more challenging than anticipated. Despite decades of intensive investigation, treatments resulting in a consistent and permanent reversal from an oncogenic cell state have remained elusive, and patient outcomes have failed to considerably improve. Although there are numerous factors contributing to cancer patient outcomes, two ever-critical problems remain, the design of more effective treatment regimes and the identification of biomarkers for patient diagnosis and prognosis. With the increasing heterogeneity and complexity observed in cancers, the need for specific and accurate diagnosis of disease state, as well as molecular monitoring of disease progression have become more important than ever. Fortunately, the intensive search for cancer biomarkers, and potential novel therapeutic targets, has revealed a factor with significant potential, the exosome. Exosomes are nanovesicles released from many cell types, but are secreted in substantially higher concentrations from cancer cells (1–3). Microvesiculation, the process of exosome production, results in the formation of small membrane-derived vesicles up 100 nm in diameter (and of a homogenous shape and density) that are released into the cellular environment by the process of exocytosis (4). They possess complex lipid membranes that contain integral proteins, while the interior cargo is comprised of an assortment of proteins and nucleic acid. These nanovesicles can travel to distant tissues where they fuse with cell membrane of target cells and induce an array of changes. In recent years, these once-neglected particles have been analyzed to reveal roles in many cellular functions, particularly cancer (5–8). This review serves to discuss the recent research on the multifunctional way exosomes affect the cellular microenvironment, and the relevance of these processes in tumorigenesis and metastasis.

**Figure 1:**
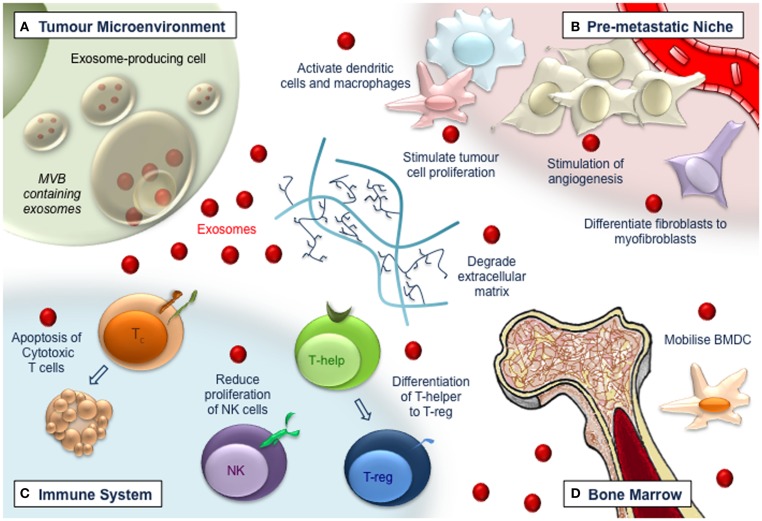
**Exosomes (red dots) have multiple roles in tumorigenesis**. **(A)** Exosomes released from tumor cells affect the local tumor microenvironment, remodeling extracellular matrix, and promoting vasculogenesis and tumor cell proliferation. **(B)** Exosomes travel to distant sites to promote the generation of the pre-metastatic niche. Vascularization is augmented and endothelial and stromal cell differentiation is induced, leading to a pro-tumor environment. **(C)** Immune responses become deregulated in a manner that impedes tumor recognition and anti-tumor immune functions. Cytotoxic T-cells are induced to apoptose, while NK cell proliferation is impaired and T-helper cells differentiate toward a T-regulatory cell phenotype. **(D)** Bone-marrow-derived cells are recruited to tumor and pre-tumor tissue where they contribute to cancer development.

Originally designated as a mechanism for the cellular release of waste and toxins, there is now substantial data demonstrating exosomes as important mediators of extracellular signaling, via the membrane-protected transfer of cellular material. Exosomes derived from both normal and malignant cells, have now been recognized as important in tumorigenesis, apoptosis, and chemotherapeutic resistance. At this stage, the contribution of exosomes to tumorigenesis primarily from two complementary processes; the modulation and restructuring of the cellular microenvironment to generate the metastatic niche (9, 10), combined with the attenuation/modulation of tumor immune responses (11, 12). In these cases, exosomes from a range of cells, induce microenvironmental changes in tissue that facilitate tumor formation, while simultaneously disarming anti-tumor immune responses, allowing cancerous cells to migrate, avoid immune detection, attach to secondary sites within the patient, and establish metastatic growth (Figure 1). Studies have revealed the significant clinical potential of exosomal signaling, both as a point of intervention or biological target in the treatment of carcinoma and prevention of chemotherapeutic resistance, as well as a potential biomarker for cancer diagnosis and prognosis. This potential has not gone unnoticed and has resulted in a substantial body of work investigating tumor-derived exosome signaling; research which is paramount in improving patient outcomes.

## The Exosomal Protein Cargo

Proteins trafficked by exosomes from both patient samples and *in vitro* cell lines have been studied in considerable detail. Studies show exosomes contain a vast array of proteins including membrane trafficking and cytoskeletal proteins, major histocompatibility complexes, signal transducers, heat-shock proteins, as well as many others (13). To date over 4,000 different proteins have been identified from purified exosomes (14). Some of these proteins are universal to most exosomes, including Tumor suppressor gene 101 (Tsg101), Heat-shock protein 70 (Hsp70), and the tetraspanins CD9, CD63, and CD81, while others seem to be specific to cell and tissue type. Some cargos suggest a role for exosomes in transformation with the identification of factors involved in cell cycle regulation, p53, epidermal growth factor (EGF), and fibroblastic growth factor (FGF) signaling, as well as angiogenesis.

The cargo of exosomes is particularly interesting as exosomes excreted from one cell are known to be able to fuse with surrounding cells, and thus have the potential to initiate signaling responses (15). This is of particular relevance in tumorigenesis, as both surrounding and distant tissues are known to adopt characteristics of the primary tumor. Many studies have now demonstrated the pertinence of exosomal proteins in cell function with particular interest paid to the role of these proteins in cancer. These proteins include the MET oncoprotein, mutant KRAS, and Tissue Factor, which are known to promote proliferation and coagulation, important processes in tumor formation. (16–18). Interestingly, the tumor environment itself may promote exosome up-take by cells. The double-layer lipid membrane structure of exosomes can change composition and rigidity in response to decreasing pH, a characteristic common to the hypoxic tumor microenvironment (15). This change in lipid composition was predicted to increase the ability of exosomes to fuse with neighboring cells. These data provide the intriguing possibility that exosomes are critical components of our signaling networks that function in the disease state to negatively affect the surrounding cellular environment.

## Nucleic Acid Trafficking via Exosomes

Tumor-derived exosomes have been shown to contain a wide range of nucleic acids, with most studies investigating exosomal microRNA or messenger RNA (mRNA). Exosomes containing these nucleic acids have been shown to be able to transfer their cargo to the recipient cells and to induce phenotypic changes (19–21). Research has revealed that tumor-derived exosomes contain distinct microRNA profiles in many cancers, including prostate (22), lung (23), breast (24), and ovarian (1). Several studies have also demonstrated the transfer of onco-microRNAs to target cells, with these microRNAs capable of modulating target pathways in host tissue (25–28). These studies have shown that oncogenic functions of microRNA may arise from the expression of both pro- and anti-tumor microRNAs. Combined with the plethora of recent work on microRNAs, it is almost certain that exosomes are fundamentally required for microRNA transport and signaling, a process particularly important in cancer.

The functional transfer of mRNA and DNA in nanovesicles has also been observed experimentally. Analysis revealed exosomes contained mutated mRNA transcripts and DNA fragments, which may contribute to growth and proliferation of many primary and metastatic cancers (29–32). A body of research has now demonstrated that tumor-derived exosomes contain a range of nucleic acids that can induce cellular responses in recipient transformed or untransformed cells. Although studies have demonstrated that many cancers express distinct RNA profiles within exosomes and that these profiles often reflect disease state, the subtle and collaborative effects of microRNA signaling and function become complicated when combined with diverse signaling potential of the other factors contained within those exosomes.

## Metastatic Niche Formation Requires Exosomes

The metastatic spread of tumor cells from the primary site, to other tissues within the body, is the primary cause of cancer mortality, but remains the most poorly understood aspect of carcinogenesis. Continuing to build on Paget’s (33) “seed and soil hypothesis,” the concept of metastatic niche formation at secondary sites is well established, thanks to an important body of work demonstrating distinct oncogenic changes including extracellular matrix restructuring and the recruitment of pro-tumorigenic factors at future metastatic sites (33). Normal cells within the microenvironment of the tumor are known to influence metastatic behavior, and there is evidence demonstrating that the successful formation of a metastatic deposit depends on the prior priming of the site for future metastatic growth (34).

It is now well accepted that primary tumor cells release a variety of cytokines and growth factors that firstly mobilize bone-marrow-derived cells and then recruit them to the site of future metastasis, creating the permissive environment for incoming tumor cells, that is called the pre-metastatic niche (10, 34). In addition to the release of soluble mediators as individual molecules, tumor cells release exosomes containing complex mixtures of these molecules, including many known effectors of tumorigenesis (13). Several studies suggest that exosomes educate bone-marrow-derived cells recruited to the pre-metastatic niche, to induce a phenotype that supports tumor cell metastasis. It was recently reported that MET receptor positive exosomes from highly metastatic melanomas could reprogram BMDCs in lung to a pro-vasculogenic phenotype (35). Early events in pre-metastatic niche formation have also been associated with exosomes, including enhanced lung endothelial permeability, proliferation, and angiogenesis, which are known contributors to tumor formation (36–38).

*In vivo* tracking of labeled melanoma exosomes injected intravenously into mice has shown that circulating exosomes are rapidly taken up from the systemic circulation into tissues, including the liver, lung, kidney, and spleen (39). Exosomes derived from renal cancer stem cells were found to contain a range of pro-tumor factors, and mice injected with these exosomes had significantly increase levels of lung cancer metastases (40). These studies therefore support a hypothesis that in addition to the influence of exosomes and their cargoes on endothelial cells within the pre-metastatic niche, other resident cells including fibroblasts and immune cells may also be stimulated to create the environment required for successful tumor cell metastasis.

Fibroblasts are a cell-type involved in the host-tumor cell interaction and more recently the activation of fibroblasts to a myofibroblastic phenotype has been associated with exosomes and has been described as a key event in the formation of the pre-metastatic niche (41–43). Myofibroblasts are often enriched in the altered stromal environment of many solid cancers, where they are known to support tumor growth, vascularization and metastasis. Modulation of the extracellular matrix has also been attributed directly to the cargo of exosomes and this may augment tumor cell invasion within the stroma of the pre-metastatic niche. Several studies have reported the deregulation of the extracellular matrix by cancer exosomes carrying factors such as glycoproteins and metalloproteinases (44–48). Exosomes that contribute to development of the pre-metastatic niche may also originate from healthy cells within the niche itself, prior to arrival of tumor cells and/or tumor-derived exosomes (49). Although the complex interplay that dictates the generation of the *in vivo* metastatic niche is yet to be fully elucidated, there is no doubt that exosomes, secreted from both healthy and cancerous cells, are necessary mediators of niche formation. Much data now suggests that exosomes contribute to niche formation through deregulation of host immune responses targeted toward the tumors.

## Exosomes Regulate Tumor Immune Responses

As well as conditioning the metastatic niche through interaction with the stromal and matrix microenvironment, exosomes display the ability to regulate immune responses targeted toward tumor cells. A significant collection of studies has explored exosome interactions with, and production by, immune cells (50, 51). Exosomes possess the ability to disable the cytotoxic arm of immune response by inducing apoptosis in cytotoxic T-cells and reducing proliferation of Natural Killer (NK) cells (12, 52). Data also indicated that tumor-derived exosome-induced differentiation of T-helper cells to regulatory T-cells, indicating a possible mechanism of evading immune surveillance, as regulatory T-cells mediate immune tolerance to tumors, by regulating tolerance of self-antigens. It has also been demonstrated that NK cells were unable to be activated, in response to Interleukin 2-mediated blocking by tumor-derived exosomes, and that tumor-derived exosomes suppress T-cells via induction of adenosine (53, 54). Apoptosis of T-cells was also found to be inducible with exosomes from melanosomes containing Fas ligand and tumor necrosis factor-related apoptosis-inducing ligand (55, 56). Additionally, tumor-derived exosomes prepared from the ascites of ovarian adenocarcinoma were found to have immunosuppressive properties, through the down-regulation of the expression of T-cell activation signaling components (57).

Exosomes have also been shown to generate anti-tumor immune responses. NK cells release exosomes containing perforin and CD56, as well as granzyme B that have been shown to inhibit tumor growth (58, 59). Due to the contradictory tumor-promoting and anti-tumor responses more research is required to determine if exosome stimulation skews the balance toward a tumor immune escape mechanism. The most probable cause for discrepancies in studies of exosomes and immune responses is due to the inherent differences between *in vitro* and *in vivo* analyses, as accounting for the complex interplay between tumor and immune responses over the course of disease has always proved problematic. It is likely that disease progression encompasses stages where exosomes induce either/both immunostimulatory and – inhibitory functions toward tumor cells. The balance between these two responses is one of the most important factors dictating disease progression, and may also provide a mechanism by which exosomal function could be regulated for the generation of novel therapies.

## Exosomes in Cancer Therapy

The use of exosomes as a vehicle for the administration of anti-tumor compounds, either as cell-derived material or therapeutic drug, has garnered considerable attention of late. Exosomes represent bioavailable vehicles that are well tolerated, bioavailable, targetable to specific tissues, resistant to metabolic processes, and membrane-permeable. This makes exosomes ideal candidates for delivery of drugs, proteins, microRNA/silent interfering RNA (siRNA), and other molecules, that would otherwise be rapidly degraded. The potential therapeutic targeting of exosomes could take a number forms, some targeting, or modulating the intrinsic effect of exosomes in the prevention of tumorigenesis or metastasis, via interactions with tumor cells, stromal tissue, and immune cells. Other strategies aim to use exosomes to generate therapeutic effects, through their use as a vehicle for the delivery of anti-tumor agents, or priming of immune responses.

The removal of exosomes from the circulatory system is an attractive therapeutic option in mitigating the metastatic effect of exosomes. Data has shown that prevention of exosome production can inhibit tumorigenesis and a range of methods have been suggested for the inhibition of exosome production, including the targeting of microtubule assembly and stability, endosomal sorting pathways, and the use of proton pump inhibitors (6, 15, 60–62). It has also been suggested that the use of extracorporeal purification already utilized to reduce viral titers in patients may be useful as a mechanism for removal of exosomes from circulation (61). Although these processes have been shown to be effective, therapies based on modulating levels or the removal of exosomes face technical and financial challenges and are yet to be implemented clinically.

Another therapeutic avenue involves the use of exosomes to efficiently deliver cargo such as drugs, microRNA’s, and antigens to target recipient cells in order to treat tumorigenesis or metastasis. The potential use of exosomes to deliver targeted chemotherapeutics has been investigated in a number of studies (63, 64). Exosomes may also provide an opportunity to deliver tissue-targeted siRNA and microRNA’s to regulate gene expression within target cells (65, 66). siRNA containing exosomes have also been shown to cross the blood brain barrier, targeting neuronal cells and knocking down their target protein by more than 60% with little toxicity (67).

Investigation has also revealed that exosomes may be an effective option for the delivery of tumor-derived antigens, to elicit an immune response (65–67). The immunostimulatory potential of exosomes was first revealed 15 years ago, when exosomes secreted from dendritic cells were found to contain functional major histocompatibility complexes that could present tumor antigen to T-cells and induce anti-tumor immune responses in mice (68). These promising results, led to Phase I clinical trials that have demonstrated that anti-tumor immune responses can be induced using dendritic-cell-derived exosomes (69–71). This has been further extended using peptide-loaded *dexosomes* (dendritic-cell-derived exosomes) as a cancer vaccine and is now in Phase II clinical trials.

## Exosomes as Cancer Biomarkers

The need for accurate novel biomarkers is of primary importance in the detection, diagnosis, and prognosis of patients, and the burgeoning area of exosomal biomarkers shows significant potential, particularly in cancer. Exosomes may provide an excellent biomarker to monitor the emergence, progression, and prognosis of cancer, as well as the efficacy of treatment regimes. Although few, studies have revealed exosomes can be readily detected in tumor tissue and many bodily fluids, and can be found in higher concentrations, both in tumor tissue, and the serum and plasma of cancer patients (1, 72, 73). The fact that exosomes display individualized expression, that are often reflective of disease state, and can be easily detected in bodily fluids, even after extended cryo-storage, make these small nanovesicles an ideal candidate for a non-invasive biomarker of tumor progression. Along with many studies characterizing possible exosome biomarkers in cell lines, there are several reports that have identified potential exosomal biomarkers in patient samples.

The presence of nucleic acid in exosomes has been described as a biomarker in glioblastoma patients via the identification of the disease-specific EGF receptor transcript (29). In an analysis of ovarian cancer patients, eight microRNAs have been identified that can be used to distinguish between benign and malignant disease, while in melanoma patients exosomes contain high levels of the proteins Caveolin-1 and CD63 (1, 74). Other studies have described markers in exosomes from prostate cancer patients (75) and non-small cell lung cancer patients (76). Interestingly, analysis of the lipids composition of prostate cancer exosomes revealed certain lipid signatures that may also serve as candidate biomarkers (77). Though the analysis of exosomes for cancer biomarkers has revealed many possible candidates (particularly microRNA), none have demonstrated enough promise to be implemented clinically. Further investigation of the dynamic expressional profile of exosomal contents throughout tumor development and treatment, combined with improved collection methods, is needed before the clinical implementation of exosomes as a biomarker for either diagnosis or prognosis.

## Exosomes and Chemotherapeutic Resistance

The acquisition of chemotherapeutic resistance is the major contributing factor to cancer mortality. While treatment regimes can be effective in the short term, the development of metastases that are refractory to radio- and chemotherapeutic treatments are common. Although this is characteristic of a wide range of cancers, the exact mechanisms by which the tumor can evade the therapeutic induction of apoptosis and develop resistance to anticancer drugs and therapies remain unclear. Data indicates that chemoresistance is likely due to a combination of processes, including increased detoxification of the drugs within the cellular environment, an increased efflux and a reduced accumulation of chemotherapeutic drugs within the cell and an increased tolerance to or repair of DNA lesions and resistance to pro-apoptotic signals (78, 79). However, the role of exosomes and nanovesicles in chemoresistance revolves around the sequestration, transport and expulsion of chemotherapeutic drugs within/from tumor cells (80, 81). Additionally, exosomes have been shown to deliver hepatic enzymes throughout the body, and given the ability of endothelial cells to up-take circulating exosomes, this may aid in the extra-hepatic detoxification of xenobiotic drugs (82).

Drug expulsion is a likely contributor to exosomal chemotherapeutic resistance. Analyses of vesicle shedding in response to drug treatment in numerous cancer cell lines revealed a consistent correlation between vesicle shedding-related gene expression, vesicle shedding rates, and drug sensitivity (83). Furthermore, vesicles isolated from these cell lines were shown to contain doxorubicin, a clinical chemotherapeutic to which tumors often develop resistance. Exosomes released from tumor cells have also been shown to contain the platinum-based chemotherapeutic Cisplatin, potentially redirecting the drug away from the nucleus where it would normally cause DNA damage, cell cycle arrest, and apoptosis (80, 81, 84).

Exosomes have also been shown to confer chemotherapeutic resistance to non-resistant cells. P-glycoprotein is an ATP-binding cassette transporter involved in multi-drug resistance in cancer (85), and has been observed transferring from drug-resistant cancer cells to recipient cells was via microparticles (86). Interestingly, Docetaxel resistance in hormone refractory prostate cancer cells can be acquired by non-invasive cell lines via exosomes. Cells then become prone to mobilization, invasion, proliferation, and anchorage dependent growth (87). Interestingly, analysis of exosomes from patient serum before and after undergoing treatment with Docetaxel showed correlation between cellular response to Docetaxel and patient response to treatment. A recent study identified another method by which exosomes may contribute to chemotherapeutic resistance. It was observed that exosomes released from cancer cells might impede antibody and drug therapies by expressing cancer derived cell surface proteins that sequester the compound away from the target cell (88, 89). Furthermore, exosomes have been shown to reduce antibody dependent cell cytotoxicity by binding to tumor reactive antibodies (90).

## Concluding Remarks

The vast repertoire of proteins and nucleic acid that can be packaged within exosomes appears to reflect the extensive, diverse, and complex signaling potential of these nanovesicles. Only now are scientists beginning to unravel the complex roles of exosomes, and although both *in vitro* and *in vivo* data clearly demonstrate the tumor-modulating potential of exosomes, the extent to which these signaling pathways dictate tumorigenesis in patients is far from being fully understood. Regardless of the contribution, the driving force behind exosome research (the significant potential of exosomes as a non-invasive biomarker, as well as a method of drug delivery and chemotherapeutic sensitization) has lead to a substantial body of work investigating these messengers. In spite of this, there are still many technical challenges that need to be overcome before the provision of exosomes as a biomarker, biological target, or drug delivery vehicle. Exosome concentrations, though increased in cancer, are relatively tiny, and methods of exosome isolation tend to be time-consuming and can be expensive while yielding samples that require further downstream purification (though these tendencies will wane with the improving technologies that yield enriched samples via affinity capture methods). Furthermore, purification techniques may serve to enrich exosomes subpopulations, often by their surface-expressed antigens or by density, which will significantly affect results. This problem may be confounded by discrepancies commonly observed in “exosome” studies, as isolation methods and terminology can differ considerably. Perhaps the greatest challenge in the investigation of exosome function is understanding the balance between healthy and oncogenic exosomal signaling, the degree to which cancer exosomes corrupt or ablate healthy exosome signaling, and the extent to which these interactions dictate metastasis over the course of disease. What makes this particularly challenging is the complex and multifunctional nature of exosomal signaling. The ability of exosomes to concurrently/concomitantly and simultaneously signal via many forms of cellular material (proteins, RNA, and lipids) makes functional analysis difficult, and this signaling method is the primary difference between exosomal signaling and other pathways (via the secretion or regulated transport individual moieties). Again, this is complicated by the fact that exosomes have been shown to possess both anti- and pro-tumor effects. To fully appreciate the signaling potential of exosomes, studies will need to investigate the co-contribution of proteins, nucleic acids, and lipids to the observed phenotype. Dissecting and revealing the contributions of each exosomal component, and modifying this intricate signaling pathway to elicit the required therapeutic response, will undoubtedly prove the most significant challenge in the utilization of exosomes as biomarkers and drug targets. Nonetheless, these significant challenges are being undertaken by many groups showing keen scientific interest in these tumor-derived exosomes and their multiple roles and functions. It is for these reasons, and the many diverse reasons discussed in this article, that exosomes may prove to be the most useful biological effector so far identified in cancer, and may finally provide viable treatment options and biomarkers.

## Conflict of Interest Statement

The authors declare that the research was conducted in the absence of any commercial or financial relationships that could be construed as a potential conflict of interest.
